# Development and validation of a visual field cluster in retinitis pigmentosa

**DOI:** 10.1038/s41598-021-89233-0

**Published:** 2021-05-06

**Authors:** Takashi Omoto, Akio Oishi, Ryo Asaoka, Yuri Fujino, Hiroshi Murata, Keiko Azuma, Manabu Miyata, Ryo Obata, Tatsuya Inoue

**Affiliations:** 1 Department of Ophthalmology, University of Tokyo Graduate School of Medicine, 7-3-1 Hongo, Bunkyo-ku, Tokyo, 113-8655 Japan; 2 Department of Ophthalmology and Visual Sciences, Kyoto University Graduate School of Medicine, Kyoto, Japan; 3 Department of Ophthalmology and Visual Sciences, Nagasaki University, Nagasaki, Japan; 4 Department of Ophthalmology, Seirei Hamamatsu General Hospital, Shizuoka, Japan; 5 Seirei Christopher University, Shizuoka, Japan; 6 Department of Ophthalmology, Shimane University Faculty of Medicine, Izumo, Japan; 7 Department of Ophthalmology and Micro-Technology, Yokohama City University, Kanagawa, Japan; 8 Nanovision Research Division, Research Institute of Electronics, Shizuoka University, Shizuoka, Japan; 9 The Graduate School for the Creation of New Photonics Industries, Shizuoka, Japan

**Keywords:** Retinal diseases, Quality of life, Glaucoma, Diseases, Eye diseases, Hereditary eye disease

## Abstract

The aim was to establish and evaluate a new clustering method for visual field (VF) test points to predict future VF in retinitis pigmentosa. A Humphrey Field Analyzer 10-2 test was clustered using total deviation values from 858 VFs. We stratified 68 test points into 24 sectors. Then, mean absolute error (MAE) of the sector-wise regression with them (S1) was evaluated using 196 eyes with 10 VF sequences and compared to pointwise linear regression (PLR), mean sensitivity of total area (MS) and also another sector-wise regression basing on VF mapping for glaucoma (29 sectors; S2). MAE with S1 were smaller than with PLR when between the first-third and first-seventh VFs were used. MAE with the method were significantly smaller than those of S2 when between the first-sixth and first-ninth VFs were used. The MAE of MS was smaller than those with S1 only when first to 3rd and first to 4th VFs were used; however, the prediction accuracy became far larger than any other methods when larger number of VFs were used. More accurate prediction was achieved using this new sector-wise regression than with PLR. In addition, the obtained cluster was more useful than that for glaucoma to predict progression.

## Introduction

Retinitis pigmentosa (RP) is a progressive hereditary retinal dystrophy in which degeneration of retinal photoreceptors causes nyctalopia and progressive visual field (VF) defects^1–3^. To evaluate disease progression, VF tests, especially in the central area, such as the Humphrey Field Analyzer (HFA; Carl Zeiss Meditec AG, Dublin, CA, USA) 10-2 tests often are used^3–5^. This measurement is important not only because it is essential to assess disease stage, but also the results are associated closely with vision-related quality of life as confirmed in RP^6^. In clinical settings, future VFs often are predicted using measured mean deviation (MD) values (MD trend analysis). However, MD reflects the values in the total area and, hence, focal VF change cannot be assessed. In contrast, pointwise linear regression (PLR) has the advantage that VF can be predicted at each test point. However, prediction often is not accurate particularly when short VF series are used^7–9^, because measurement noise is considerable even with good reliability indices^10,11^ and also because short-^12^ and long-term VF sensitivity fluctuations occur^13^. Cluster-wise regression is a compromise between these two methods^14–16^.

We previously reported that this approach is advantageous when predicting future VFs with the HFA 24-2 test compared to PLR in glaucoma cases^17,18^. Indeed, cluster analysis is in clinical use, having been adopted in Octopus 900 EyeSuite software (Haag-Streit, Inc., Köniz, Switzerland)^19^. However, to our knowledge, no method exists to cluster VF (HFA 10-2 test) in RP. We established a new VF cluster for RP with the HFA 10-2 test, and investigated whether this approach was useful to improve prediction accuracy when predicting future VF.

## Results

Demographic data for the training and testing datasets are shown in Tables 1 and 2, respectively. Mean age ± standard deviation (SD) and mean MD values at the first VF were 51.3 ± 15.7 years and − 17.9 ± 10.5 dB, respectively, for the training dataset, and 47.7 ± 12.1 years and − 16.4 ± 8.0 dB, respectively, for the testing dataset. The observation period between the first and 10th VF was 8.1 ± 1.9 years.

**Table 1:** Demographic data of the patients for the cluster developing.

Variables	Values
Eye, right:left	426:432
Age, mean ± SD, y	51.3 ± 15.7
MD, mean ± SD, dB	− 17.9 ± 10.5

*SD* standard deviation, *MD* mean deviation.

**Table 2:** Demographic data of the patients for the validation.

Variables	Values
Eye, right:left	100:96
Age at first VF, mean ± SD, y	47.7 ± 12.1
Interval between first and 10th VFs, mean ± SD, y	8.1 ± 1.9
MD (first VF), mean ± SD, dB	− 16.4 ± 8.0
MD (10th VF), mean ± SD, dB	− 20.8 ± 8.6

*VF* visual field, *SD* standard deviation, *MD* mean deviation.

**Figure 1:**
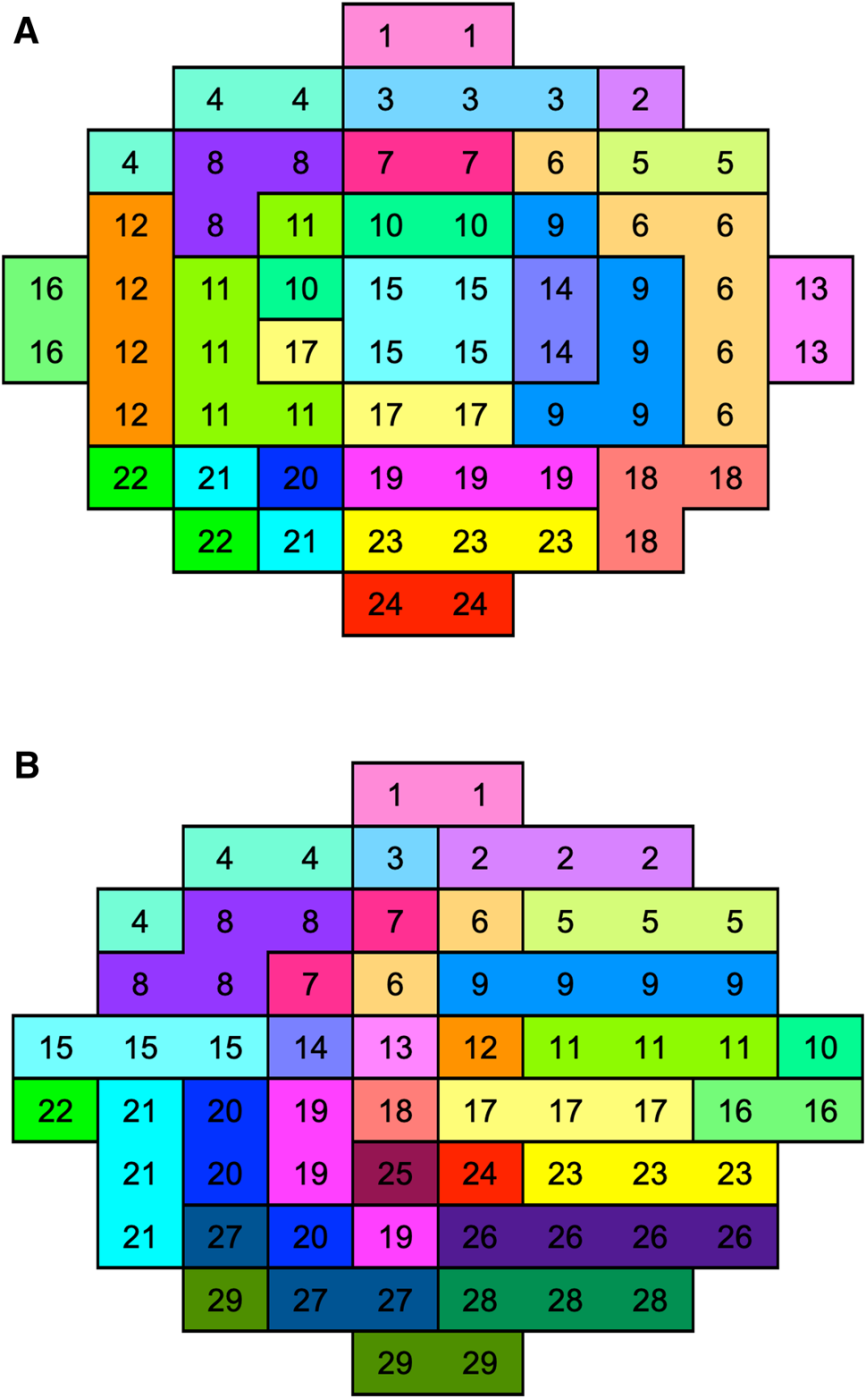
Cluster mappings of the HFA 10-2 test (right eye). The 68 test points of the HFA 10-2 test were stratified into 24 sectors (**A**: S1). The sectors of S2 mapping (**B**: 29 clusters) was derived from previous study in glaucoma^16^. The left eye was mirror imaged.

The 68 test points in the HFA 10-2 test were clustered into 24 sectors (S1 mapping; Fig. 1A) using the Hierarchical Ordered Partitioning and Collapsing Hybrid (HOPACH)—Partitioning Around Medoids (PAM) algorithm^20^ for the training data. VF mapping for glaucoma (29 sectors: S2 mapping; Fig. 1B) established with the same method in the former study^16^ were also used for comparison.

**Figure 2:**
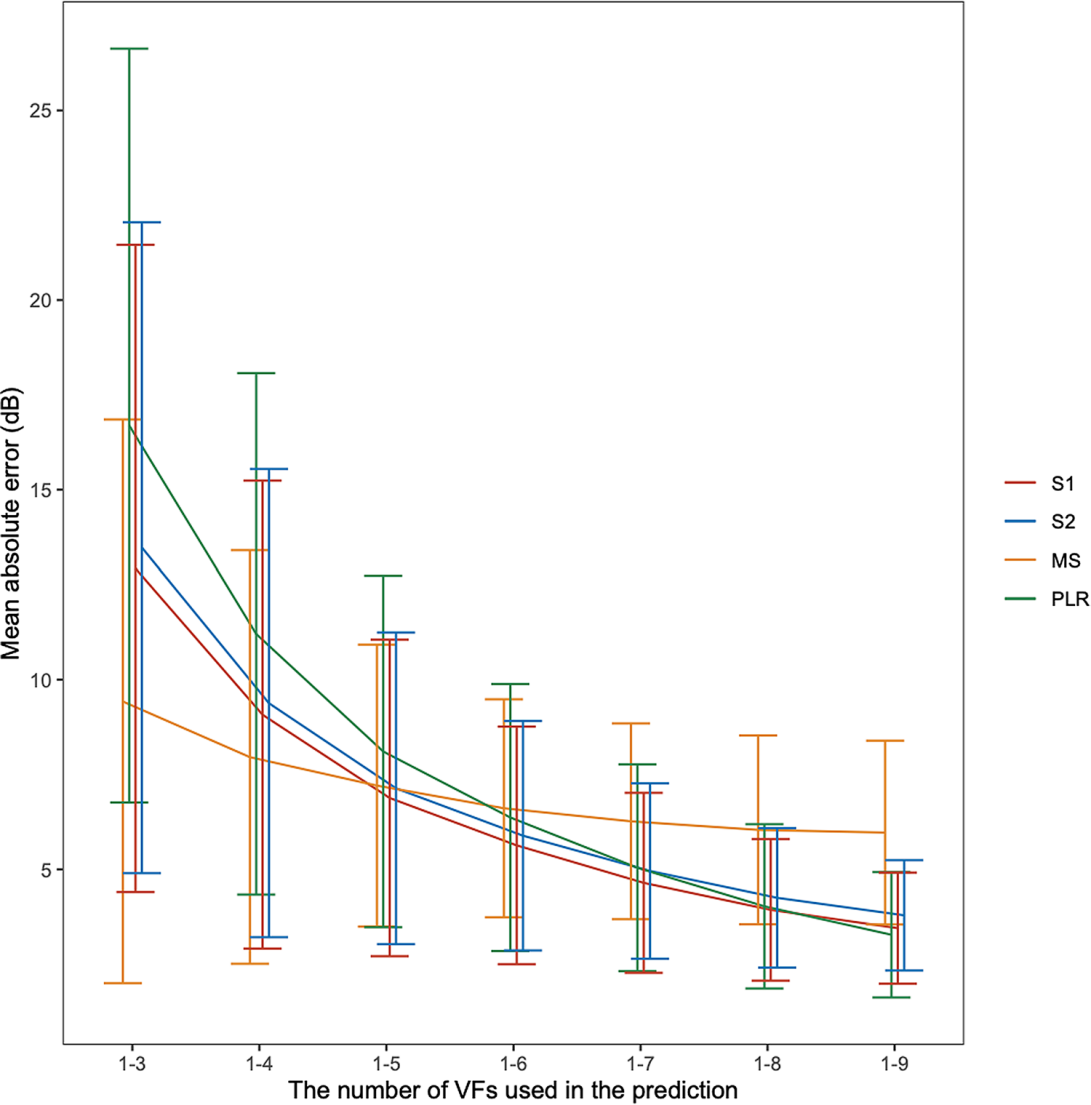
Mean absolute error when predicting the 10th VF. MAE values associated with sector-wise regression (S1 mapping) were significantly smaller than those with PLR, when the first three to first seven VFs were used, and significantly smaller than those with the S2 mapping when the first six to first nine VFs were used. Data are expressed as mean ± SD.

Figure 2 and Table 3 show the mean absolute error (MAE) values when predicting the total deviation (TD) values in the 10th VF. MAE values associated with sector-wise regression (S1 mapping) were significantly smaller than those with PLR, when the first three to first seven VFs were used (*P* < 0.001, linear mixed model adjusted for multiple comparisons using Benjamini and Hochberg’s method^21^). MAE values with these two methods were not significantly different when the first eight VFs were used (*P* > 0.05). This value was significantly larger with the sector-wise regression (S1 mapping) than with PLR when the first nine VFs were used (*P* = 0.0097). MAE values with sector-wise regressions (S2 mapping) were significantly smaller than those with PLR when the first three to first six VFs were used (*P* < 0.001). This value was not significantly different when the first seven VFs were used (*P* > 0.05). In contrast, this value was significantly larger than that with PLR in other longer (first eight or nine) VF sequences (*P* = 0.0059 and *P* < 0.001 when the first eight and first nine VFs were used, respectively). MAE values with sector-wise regressions (S1 mappings) were significantly smaller than those with the S2 mapping when the first six to first nine VFs were used (*P* = 0.021 and *P* < 0.001 when the first six and the longer sequences were used, respectively). There was no significant difference otherwise. The MAE of mean sensitivity of total area (MS) was smaller than those with S1 only from 1st to 3rd to 1st to 4th VFs were used. In contrast, the prediction accuracy became far larger than any other methods when larger number of VFs were used.

**Table 3:** Mean absolute error when predicting the 10th VF.

	VF sequences	1–3	1–4	1–5	1–6	1–7	1–8	1–9
MAE (dB)	S1	12.9 ± 8.5	9.1 ± 6.2	6.9 ± 4.2	5.6 ± 3.1	4.6 ± 2.4	3.9 ± 1.9	3.4 ± 1.5
	S2	13.5 ± 8.6	9.4 ± 6.2	7.1 ± 4.1	5.9 ± 3	5.0 ± 2.3	4.2 ± 1.8	3.8 ± 1.5
	PLR	16.7 ± 9.9	11.2 ± 6.9	8.1 ± 4.6	6.4 ± 3.5	5.0 ± 2.7	4.0 ± 2.2	3.3 ± 1.7
	MS	9.4 ± 7.4	8.0 ± 5.4	7.2 ± 3.7	6.6 ± 2.9	6.3 ± 2.6	6.0 ± 2.5	6.0 ± 2.4
*P* values	S1 vs. PLR	< 0.001*	< 0.001*	< 0.001*	< 0.001*	< 0.001*	0.22	0.0097*
	S2 vs. PLR	< 0.001*	< 0.001*	< 0.001*	< 0.001*	0.38	0.0059*	< 0.001*
	S1 vs. S2	0.16	0.29	0.22	0.025*	< 0.001*	< 0.001*	< 0.001*
	S1 vs. MS	< 0.001*	0.0011*	0.22	< 0.001*	< 0.001*	< 0.001*	< 0.001*
	S2 vs. MS	< 0.001*	< 0.001*	0.76	< 0.001*	< 0.001*	< 0.001*	< 0.001*
	PLR vs. MS	< 0.001*	< 0.001*	0.0011*	0.22	< 0.001*	< 0.001*	< 0.001*

*P < 0.05.

*VF* visual field, *MAE* mean absolute error, *PLR* pointwise linear regression, *MS* mean sensitivity.

## Discussion

We developed a new clustering method for the HFA 10-2 test in RP. As a result, 68 test points were stratified into 24 clusters. The mapping obtained was considerably different from that in glaucoma (29 VF clusters), reflecting the difference in disease mechanisms. We also validated the usefulness of the cluster-wise regression based on mapping when predicting future VF. As a result, this approach enabled far more accurate prediction than PLR, in particular when the number of VF was small. The prediction accuracy was better than when the VF cluster for glaucoma was used.

Few studies have investigated the test–retest reproducibility of pointwise VF sensitivity in RP. However, such studies in glaucoma have shown that reproducibility is poor^22^ and as a result, PLR can be associated with a considerably high false-positive rate^7^. A possible approach to overcome this problem could be reducing the variability by dividing the VF into subsectors^23^ and predicting progression based on this approach. Our study suggested that the MAE values associated with this new sector-wise regression model were significantly smaller than with PLR when between the first three and seven VFs were used. As described above, the prediction accuracies of cluster-wise regression and PLR are balanced between the variability of pointwise VF sensitivity and the masking effect of taking the average in sectors. Reflecting this point, PLR showed better prediction accuracy than the proposed cluster-wise regression when the first nine VFs were used, although no significant difference was observed between the proposed cluster-wise regression and PLR when the first eight VFs were used. On the other hand, the MAE of MS was smaller than those of sector-wise regression (both S1 and S2) when first to 3rd and first to 4th VFs were used, whereas the prediction accuracy became far larger than any other methods when larger number of VFs were used. We measured VF with an approximately 0.8-year interval. In other words, the cluster-wise regression was more useful than PLR for the duration of approximately 5 years (0.8 × 6 years).

To date, limited treatment options exist for RP. However, various attempts have been made. Vitamin A is such an example. However a recent meta-analysis suggested that treatment outcomes varied widely across studies^24–31^. A reason for contradicting results would be the different VF assessments across the studies^31^. The current results would be relevant when assessing the effect of such possible treatment using point-wise VF progression with HFA 10-2 test.

Kinetic perimetries, such as Goldmann perimetry, is another useful method to evaluate VF in the periphery in particular. However, quantitative assessment is not a primal/usual method or purpose with this type of perimetry. MD trend analysis also is used frequently, but it is not ideal to detect focal and early progression because the value reflects the total area. PLR may be a good solution, but it often is inaccurate particularly when a short VF series is used^7–9^, because measurement noise is considerable^10,11^. Our results will be useful to assess the effects of any candidate treatment on the progression of VF with the HFA 10-2 test in a relatively short duration (such as up to 5 years). In addition, vision-related disability in daily life is associated closely with the results of HFA 10-2 testing in RP^6^. Therefore, our results also will be useful when predicting patient future disability.

S1 (RP) and S2 (glaucoma) clusters have similar cluster numbers (24 and 29). However, there are considerable differences in mappings. As we described previously^16^, S2 was mapped following the retinal nerve fiber layer (RNFL), reflecting the disease mechanism. For instance, Weber et al.^32^ reported a preserved “central isle” of the VF in advanced glaucoma patients and Hood et al.^14^ suggested that this is because the RNFLs in this area penetrate the optic disc margin at the temporal angle, which usually is less likely to be affected in cases of early-to-moderate glaucoma. As a result, test points in this region tended to be clustered in a sector (sectors 11, 16, and 17 in S2). This finding was not observed in S1; S1 was simply concentric, similarly to our prior findings^33^. In addition, there was no cluster in S2 crossing the horizontal line, reflecting the distribution of RNFL. In contrast, most clusters near the horizontal line in S1 crossed the horizontal line (sectors 6, 9, 11, 12, 12, 14, 15, and 16), because the disease mechanism of RP is in retinal photoreceptors. This difference would have contributed to the better prediction accuracy of the cluster-wise regression based on S1 compared to that based on S2.

We previously compared the prediction performance of cluster-wise regression based on different VF mapping for glaucoma with large (fewer cluster numbers, 10) and small (fewer cluster numbers, 23) clusters. As described previously, the prediction accuracies of cluster-wise regression and PLR are balanced between the variability of pointwise VF sensitivity and the masking effect of taking the average in sectors. As a result, we showed that the prediction performance was better when small clusters were used in general. However this tendency was more obvious when small number of VFs were used^17^. In contrast, significantly smaller MAE values were observed with S1 compared to S2 when relatively longer VF series were used (from the first six to first nine VFs were used; Fig. 2), despite the larger clusters in S1 than in S2. This implies that the reason for the better prediction accuracy with the proposed cluster-wise regression based on S1 compared to that based on S2 is not because of the cluster size, but because the nature of VF damage in RP is better reflected in S1.

In addition, it may be advantageous to change the schedule of the VF measurement. For instance, Crabb and Garway-Heath et al. have reported the usefulness of ‘wait-and-see’ approach in which VF measurements were intensively carried out at baseline and end of the observation period, rather than with a regular interval, resulted in an improved detection rate with fewer false-positives of progression^34^. However, it has not been investigated whether this approach was also useful in the prediction of future VF as in the current study. Moreover, this approach is useful when monitoring VF progression in a fixed duration, such as in randomized control trials, however VF is usually followed up without deciding the ‘end date’ in clinics in the real world. Furthermore, according to a previous study investigating on VFs of glaucoma patients, it is useful to truncate sensitivities below 15–19 dB^35,36^. However, we did not adopt this method, because it has not been used at clinical settings, such as in the Glaucoma Progression Analysis software (Carl Zeiss Meditec AG, Jena, Germany) or PROGRESSOR tool (Medisoft, London, UK).

One limitation of the study is that genetic information could not be considered. For instance, variants in many genes, including *P23H*^37^, *PRPH2*^38^, and *PROM1*^39,40^ genes, are associated with various phenotypes. It would be interesting to investigate the influence of genetics on VF mapping, which would be particularly important when identifying individuals who could benefit from retinal gene therapy^41^, even though there might be different progression rate within the same family. Second, we could not evaluate results with longer follow-up as in our previous study on glaucoma^18^, because of the shortage of such long VF series. This should be investigated in a future study. In the current study, clustering was performed using the HOPACH-PAM method^20^, however different results may be obtained when different clustering method is applied, such as the k-means in conjunction with Dt contingency^42,43^. Furthermore, peripheral (or full field) visual field with HFA 30-2 or 24-2 test results could not be collected in the study. Clustering in wider field area should be investigated in a future study.

In conclusion, a novel VF cluster was developed specifically for RP. A cluster-wise regression based on this mapping enabled accurate prediction of future VF.

## Methods

This study was approved by the research ethics committee of the Graduate School of Medicine and Faculty of Medicine at the University of Tokyo and Kyoto University, and complied with the tenets of the Declaration of Helsinki. Written informed consent was given for patient information to be stored in the hospital database and used for research. Otherwise, based on the regulations of the Japanese Guidelines for Epidemiologic Study 2008, the study protocols did not require that each patient provide written informed consent. Instead, the protocol was posted at the outpatient clinic and/or website of the department to notify study participants.

### Participants

This retrospective study included 858 eyes of 442 patients with RP obtained at the retinal clinic at Kyoto University Hospital. All patients underwent at least two reliable HFA 10-2 tests (SITA-standard program). The first examination was excluded from the study. An unreliable VF was defined as having ≧ 20% fixation losses or ≧ 15% false-positive errors, following manufacturer’s recommendations. After exclusion, eyes with ≧ 10 VF records (196 eyes/103 patients) were used as the testing dataset and the remaining 858 eyes (442 patients) were used as a training dataset. The training dataset was used to develop the VF cluster in RP. Only the initial VFs were used when a patient had ≧2 VFs. The testing dataset was used to validate the usefulness of the cluster-wise regression. Only the initial 10 VFs were included when a patient had > 10 VF test results.

Inclusion criteria were: (1) typical fundus findings of RP, such as bone spicule pigmentation, arteriolar attenuation, and waxy disc pallor; (2) reduction in a- and b-wave amplitudes or nondetectable full-field electroretinogram; (3) RP was the only disease causing VF damage; (4) no previous ocular surgery except for cataract extraction and intraocular lens implantation; (5) no other diseases of the anterior and posterior eye segments that could affect VF, including cataracts, except for clinically insignificant senile cataracts; and (6) age at least 20 years. Those with intraocular surgery, including cataract surgery, during the observation period were excluded.

### VF sectors

The 68 test points in the HFA 10-2 test were clustered using HOPACH-PAM algorithm^20^. The details of this method have been reported previously^16^. In brief, the method is a hybrid between hierarchical ordered partitioning and collapsing, whereby a hierarchical tree is built by recursively partitioning a data set, while ordering and possibly collapsing clusters at each level. HOPACH builds a hierarchical tree of clusters by recursively partitioning the data, while ordering and possibly collapsing clusters at each level to identify finite structures in a dataset using the Mean Median Split Silhouette criteria^20,44^. A strength of this approach is that the optimum number of clusters is inferred automatically^20^, unlike many other clustering methods, such as k-means and hierarchical clustering methods, where the number of optimum clusters is decided using additional statistical analyses to determine their separability.

### Statistical analysis

Following creation of the VF cluster (S1 mapping; Fig. 1A), mean absolute error (MAE) of the cluster-wise regression was evaluated using the testing dataset, as follows: (1) 68 test points were stratified into 24 sectors for each of the initial three VFs. (2) In each sector, mean TD values were calculated and allocated to all test points belonging to the sector. (3) The 68 TD values of the 10th VF were predicted by extrapolating the allocated TD value against time at each test point. These predictions of 10th VFs were iterated using other VF sequences (from first-fourth to first-ninth VFs). For comparison, similar calculations were conducted using the PLR, MS and also another sector-wise regression basing on a VF mapping for glaucoma (29 sectors: S2 mapping; Fig. 1B)^16^. These MAE values were compared among the methods using the linear mixed model, in which the subject was the random effect^45,46^. Benjamini and Hochberg’s method was used to adjust for multiple comparisons^21^.

All analyses were conducted using R software version 3.5.2. (the R Foundation for Statistical Computing, Vienna, Austria).
